# Editorial: Humanitarian Activities in Pediatric Cardiology

**DOI:** 10.3389/fped.2021.758247

**Published:** 2021-10-04

**Authors:** Antonio F. Corno, Sanjiv Nichani

**Affiliations:** ^1^Children's Heart Institute, University of Texas Health, Houston, TX, United States; ^2^Healing Little Hearts, Charity Association, Leicester, United Kingdom

**Keywords:** humanitarian, cardiology, pediatrics, integrated healthcare, cardiac program

Several studies have reported, in emerging economies, an incidence of congenital heart defects that is higher than average because of several factors, including genetics, high prevalence of consanguineous marriages, malnutrition, poor sanitation, and high fertility rates (1). Congenital malformations, of which heart defects represent nearly 50%, are the 4th leading cause of neonatal mortality. Since the care of patients with congenital heart defects requires significant resources, the main global issue remains that access to care is not equal for all (1). As a result, every year about 90% of over one million of children with congenital heart defects, predominantly born in low-income countries, do not have access to care or only receive sub-optimal care (1). This tragic situation has stimulated the creation of many non-profit humanitarian organizations, trying to reduce the imbalance existing for children across the globe. The purpose of this Research Topic was to share the experience of people involved in humanitarian activities in emerging economies in different locations of the world, combining different perspectives: anesthesists, surgeons, nurses, entire teams with long standing activity in one hospital, and also the recipients of one of these programs.

Jivanji et al. presented very clearly the situation in sub-Saharan East Africa: Kenya, Tanzania, and Uganda, with their commentaries on affordability, access, and awareness. They listed all challenges faced in developing a cardiac program in that region, with problems such as corruption at various levels described with a very clear narrative (Jivanji et al.).

Giamberti et al. presented their remarkable experience over a 17-year period of creating and developing a pediatric cardiac unit in Cameroon, central Africa, with accurate description of barriers and facilitators encountered for the set-up. This team not only organized the clinical activities but also a fruitful bilateral exchange of collaboration with the local caregivers, with continuous educational programs, and constant monitoring of the progress made. The most successful result is that this unit, the only one active in the entire country of Cameroon, is now attracting patients from surrounding countries (Giamberti et al.).

Cvetkovic reported her experience gained in various visits to India, Malaysia, Nigeria, Kenya, Tanzania, and Mauritius. Thanks to the opportunity of experiencing different environments, she presented her approach to improve the quality of anesthesia derived from the combination of the following: regular teaching and education sessions, careful patients selection, patients preparation for surgery with meticulous pre-operative care, individualized type of anesthesia, and structured daily team briefing (Cvetkovic). The results were very encouraging, particularly because every visit was followed by regular interaction between visiting and local teams (Cvetkovic).

Howes, a specialist nurse with years of experience in Pediatric Cardiac Intensive Care Unit who was involved in many visits with different teams, presented her interesting perspective regarding the protocol of early extubation after repair of complex congenital heart defects. Advantages and disadvantages of early extubation were analyzed, balancing the potential risks (lung collapse, respiratory insufficiency, re-intubation) vs. the needs dictated by limited resources in terms of personnel, equipment, infrastructures. Most of these aspects can be taken into consideration in different types of environments (Howes).

A relatively new perspective has been presented by Zain et al., who reported the experience of the local team in a rural area of North-East Malaysia, exposed to the beginning of pediatric cardiac surgery activities from scratch, initially with a surgeon in place for a couple of years, then with regular visits of alternating teams. Their experience is another confirmation that, if a program is well-organized and sustained, it can lead to stepwise growth of the local team until reaching autonomy for surgical management of the vast majority of children born with congenital heart defects in a region without any alternative available for treatment. The visiting teams now are mostly helping the local team for assistance in the surgical management of the most complex malformations, and to continue the educational and supporting program (Zain et al.).

The last two articles of this Research Topic reviewed the entire issue of humanitarian activities in low and middle-income countries.

Novick et al. reported their approach, accomplished after mastering the assistance in pediatric cardiac surgery, starting from visiting Colombia in 1991 and since then having visited 32 countries, having completed over 560 trips of between 1- and 4-weeks in duration, with nearly 10,000 patients operated on in total. Their comprehensive approach covers many steps, including the request for assistance, a site visit, the preparation for program implementation, different types of program models accordingly with the match between the local needs and the visiting team availability (intermittent visit model, resident senior surgeon model, team in residence model), the nursing education and empowerment, and the attention to the services of interventional cardiology, anesthesia, perfusion, biomedical engineering, operating room (Novick et al.). Because of their very extensive experience across many countries, Novick et al. also analyzed the difficulties of providing service in conflict zones, the need to provide benchmarks and indicators of improvement, as well as quality improvement and assessment, and ethical considerations on the entire matter.

Murala et al. provided a detailed overview of the current global situation, with report of the current units involved in these humanitarian activities with short and long-term programs. In particular, for the short-term programs the analysis included persistence and continuity, cooperation with Non-Governmental Organizations, focus on types of congenital heart defects, on site issues, view from the host programs, and financial aspects. Then they proposed elements to consider in a model for establishing long-term programs: shared vision, spark plug (organization or individual who is a dedicated leader, focused, invested, and physically present in a local program on a long-term basis), communication and coordination, training, material support, exit strategy, data collection, and analysis (Murala et al.).

In the final article, Nichani and Nichani explored in a clever and original way the potential benefits of this work performed by teams from the so called “high income countries” to the low-income regions of Africa and Asia to provide care for children born with congenital heart defects.

In fact, there are also advantages for those teams organizing these humanitarian activities, such as gained experience on unusual cases, team-building, and improvement. Furthermore, a deeper connection to the main meaning of the medical profession is an extremely important part of the experience.

This is, to the best of our knowledge, the first time that the many benefits for the traveling medical teams, in addition to the recipients, have been considered in all the literature published on this topic.

We hope that this Research Topic will help all readers and interested individuals so that in the future the diagnosis of congenital heart defects and rheumatic heart diseases will no longer be the death sentence, for the majority of children in the world, that it currently is.

## Author Contributions

All authors listed have made a substantial, direct and intellectual contribution to the work, and approved it for publication.

## Conflict of Interest

The authors declare that the research was conducted in the absence of any commercial or financial relationships that could be construed as a potential conflict of interest.

## Publisher's Note

All claims expressed in this article are solely those of the authors and do not necessarily represent those of their affiliated organizations, or those of the publisher, the editors and the reviewers. Any product that may be evaluated in this article, or claim that may be made by its manufacturer, is not guaranteed or endorsed by the publisher.
